# Dataset for structural health monitoring of pipelines using ultrasonic guided waves

**DOI:** 10.1016/j.dib.2022.108756

**Published:** 2022-11-17

**Authors:** Mahjoub El Mountassir, Slah Yaacoubi

**Affiliations:** Equipe Monitoring et Intelligence Artificielle, Institut de Soudure, 4 boulevard Henri Becquerel, 57970 Yutz, France

**Keywords:** Ultrasonic guided waves, Structural health monitoring, Environmental conditions, Pipeline

## Abstract

Ultrasonic guided waves (UGW) technique is widely used for nondestructive testing (NDT) of pipelines. In structural health monitoring (SHM), this technique can used to continuously assess the health of these kind of structures. However, environmental conditions can affect UGW propagation and result in false indication of defects. This article describes and provides a database of UGW signals that were acquired at different temperatures during a long period of time. This database could be used to test the efficiency of temperature compensation methods. Besides, a corrosion like defect was created at the end of the monitoring period. Therefore, damage detection methods in SHM could be tested as well.

**Specifications Table**

**Table:** 

Subject	Mechanical Engineering
Specific subject area	Structural health monitoring
Type of data	Excel files
How the data were acquired	Data acquisition was ensured using Wavemaker G4 system commercialized by Guided Ultrasonics LTD.
Data format	Raw
Description of data collection	The data was collected using UGW technique (Wavemaker G4) from a pipe segment (tube) repaired locally by patch composite. This pipe was monitored during a period of almost 3 months. During the last day of the monitoring period, a corrosion-like damage was created by removing material from inside the pipe in 6 steps. In total, 236 UGW signals were collected where 207 ones from healthy state and 29 ones from damaged state.
Data source location	Data was obtained at the Institut de Soudure, Yutz, France
Data accessibility	Repository name: Mendeley platform. Data identification number: 10.17632/ttb63krg6d.1 Direct URL to data: https://doi.org/10.17632/ttb63krg6d.1
Related research article	M. El Mountassir, S. Yaacoubi, G. Mourot, D. Maquin, Sparse estimation based monitoring method for damage detection and localization: A case of study, Mech. Syst. Signal Process. 112 (2018) 61–76.

## Value of the Data


 

• UGW signals were acquired at different temperatures. Therefore, this dataset can serve to test existing temperature compensation methods or help in the development of new more robust ones.
• This database of signals can be also used to test the efficiency of defect detection methods.
• The influence of the excitation frequency on the damage detection method can be investigated.
• The sensitivity of defect detection to UGW propagation mode, either torsional or flexural, can also be evaluated.

## 1 Data Description

**Fig. 1:**
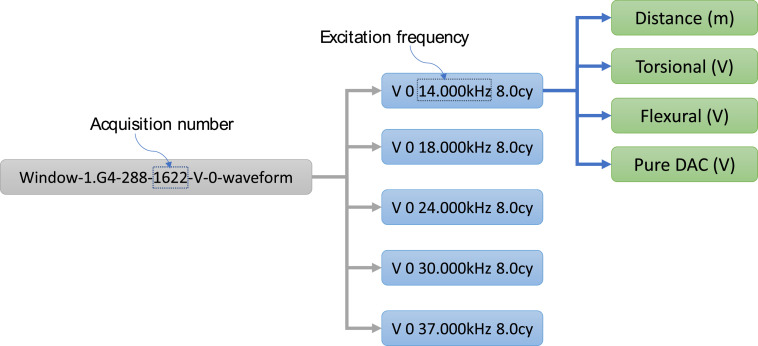
Content of an acquisition excel file.

The dataset was gathered on a pipe segment (tube). As the tube was empty, the only environmental factor that can be considered is ambient temperature. Its variation was comprised roughly between 19°C and 26°C. The monitoring period was fixed around three months. During this period, multiple UGW signals were acquired from the healthy state tube. At each acquisition, five repetitive signals were generated. During the last day of the monitoring period, damage was created artificially using a grinding machine to simulate corrosion at a distance of 2.6 m from the transducer. The dimensions of the created defect were increased randomly in six steps. Each UGW acquisition was then saved in an excel file. This file contains five sheets. Each one corresponds to a specific frequency of excitation. The used frequencies are 14 kHz, 18 kHz, 24 kHz, 30 kHz and 37 kHz. Each sheet contains a matrix of data of 2057 × 4. The lines are the number of samples obtained using a sampling frequency of 195 kHz while the columns correspond to the propagation distance, torsion mode, flexion mode and DAC (Distance Amplitude Correction) curve. It is worth noting that the last column can be omitted as all it values are null. An example of the content of an excel file is given in Fig. 1.

**Fig. 2:**
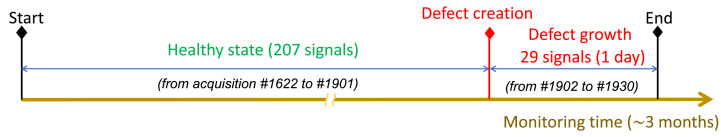
Schematic representation of the collected dataset.

Fig. 2 illustrates the followed steps to build dataset. As it can be seen, 207 signals were firstly acquired from the healthy state of the tube (from acquisition #1622 to # 1901). Then, 29 signals were collected from the damaged state (from acquisition #1902 to #1930).

## 2 Experimental Design, Materials and Methods

As mentioned before, the generation and reception of signals was ensured using UGW technique. UGW are stress waves, which propagate through an elongated medium and are guided by its boundaries [1], [2], [3]. They can propagate over long distances in structures, making it possible to detect defects over a considerable area with sparse number of transducers [4]. The transducers can be arranged by two configurations: pitch-catch and pulse-echo. In the former, a transducer can only excite or receive the UGW while in the later, the transducer is used simultaneously for the generation and reception of UGW. When the transducers are excited, UGW travel in all directions and interact with the geometric heterogeneities (i.e., damage, welds, structure ends, etc.) that can exist in their way. This propagation involves two phenomena: dispersion and multimodal behavior. As a consequence of dispersion, the travel velocity depends on the excitation frequency. Concerning the multi-modes behavior, when dealing with cylindrical structures (hole such as tube or not, like rods), three types of modes can be excited: longitudinal, torsional, and flexural [1].

**Fig. 3:**
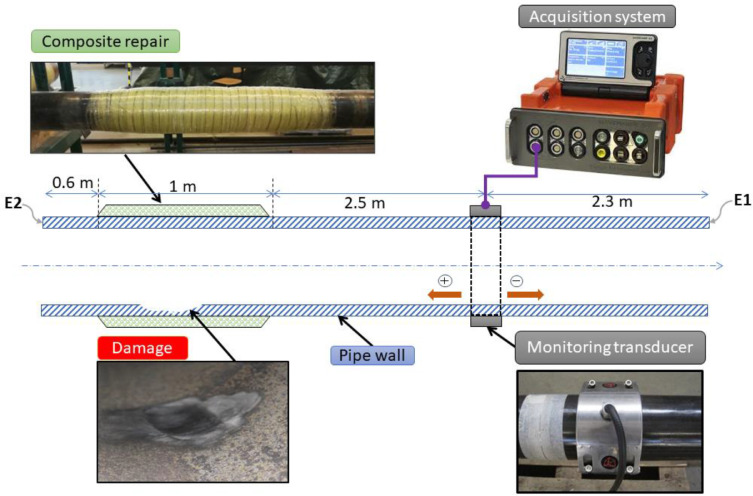
Experimental setup showing a sectional representation of the tube, the used transducer, the created damage, the composite repair, and the acquisition system.

Fig. 3 shows the experimental setup used to build the dataset. It consists of a monitoring transducer, which was mounted on a tube using pulse-echo configuration. This transducer is composed of two rows of circumferentially equidistant piezoelectric sensors. These rows of sensors allow the control of UGW propagation direction using the interference phenomena [5]. The data acquisition system, which is provided by Guided Ultrasonic Ltd company [6], was connected to the monitoring transducer. This acquisition system is only able to operate with two UGW modes, which are torsional (non-dispersive) and flexural (dispersive).

The dimensions of the tube are: length = 6.4m, external diameter = 152.4mm and thickness = 7.1mm. This tube has already served for a research study to evaluate interalia the effect of composite repair on the attenuation of UGW. The damage was created intentionally inside the tube (inner the wall of the region below the composite repair). The objective here was to investigate the capabilities of UGW to detect the initiation and growth of damage under the composite repair.

**Fig. 4:**
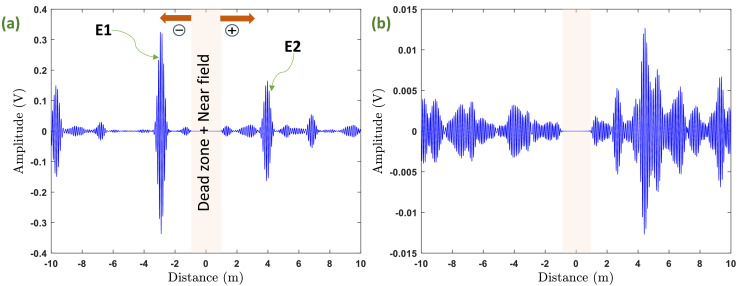
Examples of acquired signal with excitation frequency of 14 kHz, (a) torsional mode and (b) flexural mode.

Examples of acquired UGW signals for the two propagation modes are shown in Fig. 4. As it can be seen, the middle part of each signal was drawn because it represents the dead zone area (i.e., excitation signal) and the near field [2]. Therefore, damage detection in this region of the tube is impossible. It can be also noticed that the interpretation of the flexural mode signal is much more complex than that of the torsional one. Actually, in this latter, we can easily identify the echoes that correspond to each end of the tube (i.e., E1 and E2). This can be explained by the non-dispersive nature of torsional mode. However, this observation does not mean automatically that the torsional mode signals will be more favored than the flexural ones since in SHM, there is no need for visual interpretation of the acquired signals. In other words, the flexural mode signals could be more sensitive some kinds of faults than the torsional mode signals thanks mainly to its non-axisymmetric nature. Additionally, the direction of the faults with regard to that of the particular motion of flexural mode waves plays an important role in its detection. It is for all these reasons that both modes families are complementary, and so no one should be neglected if no a priori exists about the fault we are looking for.

**Fig. 5:**
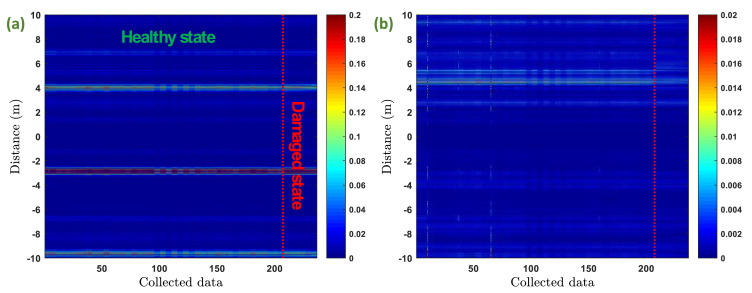
Top view representation of the collected data with an excitation frequency of 14 kHz: (a) torsional mode, and (b) flexural mode.

In order to have a global overview of the collected data for a specific excitation frequency, the signals were represented first in a 3-dimensional space, then a top view image was obtained for the two propagation modes as shown in Fig. 5.

The x-axis is the number of collected signals and the y-axis is the propagation distance. The colormap represents the amplitude of signals. This colormap was set intentionally to a limit below the maximum amplitude for both propagation modes in order to emphasize differences in amplitude between the healthy state signals and damaged state signals. It can be noticed that in both representations (i.e., for torsional and flexural modes), there is no visual clear separation between the healthy state signals and damaged state signals. Furthermore, some changes in the amplitude of the healthy state signals can be observed. These changes are probably due to the variation of temperature during the monitoring period.

Finally, note that the temperature range variation (TRV) is the current case is 7°C while it is often much larger in real life. There are some cases, although few, where the said range is not very large. A necessary condition but not sufficient to get this is the indoor monitoring, on which we focus here. Tubes are used in many factories, refineries, and covered halls. Even if the TRV is small, its impact on the diagnostic result is not negligible, and should be addressed [7]. Of course, this dataset cannot be a sufficient platform to test strategies to mitigate the effect of temperature of UGW measurement in all possible cases, but it is still useful even in the case where the TRV is large since it can serve as a first step to test the developed temperature compensation strategies (i.e., if they do not work on the current dataset, they will definitely not work in a harsher one).

## Ethics Statements

This work does not involve any ethics fields.

## CRediT authorship contribution statement

**Mahjoub El Mountassir:** Data curation, Visualization, Investigation, Writing – original draft. **Slah Yaacoubi:** Conceptualization, Methodology, Investigation, Writing – review & editing, Funding acquisition, Supervision.

## Declaration of Competing Interest

The authors declare that they have no known competing financial interests or personal relationships that could have appeared to influence the work reported in this paper.

## Data Availability

Ultrasonic guided waves data for structural health monitoring of pipelines (Original data) (Mendeley Data)
